# Protein Carriers for Glycoconjugate Vaccines: History, Selection Criteria, Characterization and New Trends

**DOI:** 10.3390/molecules23061451

**Published:** 2018-06-15

**Authors:** Francesca Micoli, Roberto Adamo, Paolo Costantino

**Affiliations:** 1 GSK Vaccines Institute for Global Health (GVGH), 53100 Siena, Italy; francesca.x.micoli@gsk.com; 2 GSK, 53100 Siena, Italy; roberto.x.adamo@gsk.com

**Keywords:** glycoconjugate vaccines, protein carriers, characterization

## Abstract

Currently licensed glycoconjugate vaccines are composed of a carbohydrate moiety covalently linked to a protein carrier. Polysaccharides are T-cell independent antigens able to directly stimulate B cells to produce antibodies. Disease burden caused by polysaccharide-encapsulated bacteria is highest in the first year of life, where plain polysaccharides are not generally immunogenic, limiting their use as vaccines. This limitation has been overcome by covalent coupling carbohydrate antigens to proteins that provide T cell epitopes. In addition to the protein carriers currently used in licensed glycoconjugate vaccines, there is a search for new protein carriers driven by several considerations: (i) concerns that pre-exposure or co-exposure to a given carrier can lead to immune interference and reduction of the anti-carbohydrate immune response; (ii) increasing interest to explore the dual role of proteins as carrier and protective antigen; and (iii) new ways to present carbohydrates antigens to the immune system. Protein carriers can be directly coupled to activated glycans or derivatized to introduce functional groups for subsequent conjugation. Proteins can be genetically modified to pre-determine the site of glycans attachment by insertion of unnatural amino acids bearing specific functional groups, or glycosylation consensus sequences for in vivo expression of the glycoconjugate. A large portion of the new protein carriers under investigation are recombinant ones, but more complex systems such as Outer Membrane Vesicles and other nanoparticles are being investigated. Selection criteria for new protein carriers are based on several aspects including safety, manufacturability, stability, reactivity toward conjugation, and preclinical evidence of immunogenicity of corresponding glycoconjugates. Characterization panels of protein carriers include tests before conjugation, after derivatization when applicable, and after conjugation. Glycoconjugate vaccines based on non-covalent association of carrier systems to carbohydrates are being investigated with promising results in animal models. The ability of these systems to convert T-independent carbohydrate antigens into T-dependent ones, in comparison to traditional glycoconjugates, needs to be assessed in humans.

## 1. Introduction

In the 1980s, glycoconjugate vaccines were introduced into the arsenal of available weapons for prevention of infectious diseases to overcome an important limitation of conventional polysaccharide (PS) vaccines [1]. Despite PS vaccines being immunogenic and protective in adults against serious infectious diseases, such as meningitis and pneumonia, they are not efficacious in the first years of life when the disease burden caused by many encapsulated bacteria is high [2,3,4]. PS are defined as T-cell independent antigens which can directly stimulate B cells to produce antibodies. They are made up of repetitive structures containing B cells but not T cell epitopes, the latter being essential to engage T cells and trigger a B-T cooperation, leading to a more complete immune response at any age [5,6]. Chemical conjugation of PS or oligosaccharide antigens to proteins results in vaccines where the protein moiety provides T cell epitopes [7]. While there are many reports on the preparation and characterization of glycoconjugate vaccines focusing on the glycan moiety, less attention has been put on aspects related to the protein carrier. Here, we provide an overview of the carriers for glycoconjugate vaccines, including traditional and more recently used recombinant proteins, exploited in some examples with the additional role of antigen. Novel carrier systems, such as inorganic nanoparticles, virus-like particles and Outer Membrane Vesicles (OMVs) are also presented. The role of protein carrier, conjugation chemistries, selection criteria, and characterization before and after conjugation are discussed. Relevant aspects related to the impact of the carrier protein and the conjugation degree on the immune response of glycoconjugate vaccines, which need to be further investigated in humans, are here analyzed.

## 2. The Concept of Hapten and Carrier

In early 20th century, Karl Landsteiner demonstrated that the specificity of the immune response to proteins (carriers) was changed when small molecules (haptens) are chemically attached to them. In particular, he found that antibodies were induced against new determinants comprising the conjugated small molecule. More interestingly, these antibodies recognized the hapten also in its native not conjugated form [8]. Later, Avery and Goebel published papers showing that the “chemical union” of small saccharides to proteins results in enhanced immunogenicity of the saccharide and that saccharide specific antibody concentrations increase after reinjection of the conjugate [9]. This series of papers culminated with the demonstration that, when the capsular PS of type III pneumococcus is conjugated to protein and then inoculated in rabbits, the rabbits produce anti-pneumococcus III specific antibodies which protect them from the challenge with a virulent pneumococcal type III strain [10].

## 3. Polysaccharide Based Vaccines

The history of PS based vaccines started with the research on vaccines against pneumococcus, focused on capsular PS decorating the surface of this pathogen. In 1932, Sutliff reported the first evidence that “protein free” preparations of pneumococcal PS elicit specific antibody titers with anti-pneumococcal power in subjects ranging in age from 15 to 56 years [11]. In 1945, the first tetravalent anti-pneumococcal vaccine was available [12].

At the same time, the introduction of antibiotics for treatments of bacterial diseases slowed down the development of PS vaccines. However, it was soon clear that antimicrobial treatment, even if successful, could not be the only solution due to treatment failures, frequent serious sequelae in subjects recovering from diseases such as meningitis, and the emergence of antimicrobial resistant strains. 

In 1978, a quadrivalent vaccine based on meningococcal serogroups A, C, W and Y PS was licensed, and three years later it was the turn of a 23-valent anti-pneumococcal PS vaccine, followed by the anti-*Haemophilus influenzae* type b (Hib) PS vaccine in 1985.

## 4. From Polysaccharide Vaccines to Glycoconjugate Vaccines

The clinical trials carried out during the development of meningococcal and *Haemophilus influenzae* type b PS vaccines showed that the efficacy in children below two years of age was very low [2]. Similar observation was done for pneumococcal PS vaccines, although with differences depending on the serotype [3], confirming some evidences already available [4]. This was a trigger point for the research and the development of glycoconjugate vaccines. The concept of carrier/hapten introduced by the works of Landsteiner, Avery and Goebel was taken up again.

The first preclinical and clinical reports on glycoconjugate vaccines were those describing Hib PS–protein conjugates by the team of John Robbins at NIH [1]. Thanks to this pioneering work, the first glycoconjugate vaccines against Hib were licensed between 1987 and 1990. At the same time, active research started in academic and industrial settings to develop conjugate vaccines against the most epidemiological relevant serotypes of pneumococcus and meningococcus, leading to licensure of many other glycoconjugate vaccines [13].

## 5. Mechanism of Action of Glycoconjugate Vaccines

PS are T-cell independent antigens capable of directly stimulating B-cell differentiation into plasma cells (which produce antibodies) by cross-linking B-cell receptors (BCR). This mechanism is not yet mature in infants where cooperation between B and T cells is required for efficient immune responses to PS immunogens [14,15]. In glycoconjugates, the protein carrier provides the T epitopes for T-cell help recruiting, while the B epitopes for PS specific B cells engagement are provided by the saccharide moiety. The latter binds to BCR and, after internalization, B cells direct the processing of the protein moiety of glycoconjugate in peptides that are presented to carrier specific T cells in association with major histocompatibility complex (MHC) class II molecules. When B cells receive T cell help, they proliferate and differentiate into plasma cells producing antibodies, with class switching particularly to IgG, and into memory B cells. The latter ones, on subsequent encounter of specific antigen, can rapidly proliferate and differentiate into plasma cells, producing high antibody titers. Antibody avidity is increased through affinity maturation in germinal centers [5]. Recently, based on studies with GBSIII-protein conjugates, an additional mechanism has been proposed according to which the glycoconjugate is processed into glycan-peptides inside PS specific B cells. The resulting glycan-peptides bind MHC class II via the peptide portion allowing the glycan moiety to be exposed and recognized by the T-cell receptor of carbohydrate specific T cell clones which then provide the cognate help to B cells [16]. According to these mechanisms, a conjugate vaccine can induce a T-cell-dependent response and be immunogenic from early infancy. It is worth of mentioning that an immunogenic response against carbohydrates has also been achieved by using glycolipid carriers, such as α-Galactosyl ceramide [17] or synthetic lipid A derivatives [18], via activation of alternative immunological pathways such as invariant natural killer T (iNKT) cell or Toll-like receptors (TLR), respectively.

## 6. Traditional Protein Carriers

Five carrier proteins are currently used in licensed conjugate vaccines: diphtheria toxoid (DT), tetanus toxoid (TT), CRM_197_, *Haemophilus* protein D (PD), and the outer membrane protein complex of serogroup B meningococcus (OMPC). DT, TT and CRM_197_ are derived from bacterial toxins detoxified by chemical or genetic means. Diphtheria and tetanus toxoids were initially selected as carriers for Hib conjugate vaccines because of their safety record established over decades of vaccination against tetanus and diphtheria. 

OMPC has been used for a Hib conjugate vaccine [19] and a first generation of pneumococcal conjugate vaccines [20]. PD is a 40 kDa cell-surface protein originally derived from non-typeable *H. influenzae* (NTH). It is produced from a recombinant strain of *E. coli* and has been introduced as a carrier for most of the serotypes in a multivalent pneumococcal conjugate vaccine [21,22]. CRM_197_ is a 58 kDa nontoxic mutant of diphtheria toxin with a single Glycine to Glutamic amino acid substitution at position 52 [23], therefore it does not require any chemical detoxification. Its crystal structure has been solved and the molecular aspects at the basis of its non-toxicity elucidated [24]. Nowadays CRM_197_ can be isolated from the supernatant of cultures of *Corynebacterium diphtheriae* C7(β197)tox(−) or produced by recombinant DNA techniques in *heterologous organisms.* Full comparability of the proteins obtained by the different manufacturing platforms has been shown [25]. CRM_197_ has been used extensively as a carrier for licensed Hib, multivalent meningococcal and pneumococcal conjugate vaccines and other vaccines in development [26,27]. 

## 7. New Protein Carriers under Investigation

Besides the proteins already established as carrier in licensed glycoconjugate vaccines, many other have been tested in preclinical studies and some also in clinical trials; a notable example is the recombinant non-toxic form of *Pseudomonas aeruginosa* exotoxin A (*r*EPA) which has been used as carrier for *Shigella O*-antigens [28], *Staphylococcus aureus* type 5 and 8 capsular PS [29], and *Salmonella Typhi* Vi antigen [30,31], and is also widely used for glycoconjugates directly synthesized in *E. coli*, known as bioconjugates [32,33,34,35]. 

In a recent study, several different recombinant proteins derived from a range of pathogens have been tested in mice as carrier for different fungal and meningococcal glycans, in comparison to CRM_197_ as bench mark. Some of them, such as the pneumococcal recombinant spr 96/2021 and spr1875, and the Extra intestinal Pathogenic *E. coli* derived Upec-5211 and Orf3526 proteins, resulted to be potential good carriers [36]. A rationally designed recombinant protein, containing strings of promiscuous human CD4^+^ T-cell epitopes derived from various pathogens including tetanus, influenza virus, *Plasmodium falciparum* and hepatitis B virus, proved to be a very good carrier for Hib and meningococcal oligosaccharides [37,38,39]. The recombinant tetanus toxin HC fragment has been used as carrier for synthetic fragments of O-PS of Vibrio cholerae O:1 [40].

As opposed to B cell epitopes that are often of conformational nature, T cell epitopes of proteins are linear sequences of a minimum of 8–12 amino acids [41] that bind to MHC class II and interact with T cell receptors on the surface of CD4^+^ T cells. Based on this, many researchers have investigated the possibility to recapitulate the complexity of protein carrier by using synthetic peptides as carrier for carbohydrate antigens. A synthetic peptide representing T-cell epitope of CRM_197_ has been investigated as carrier for Hib carbohydrate antigen [42]. One of the best known examples is the 13 amino acids non-natural pan DR epitope (PADRE) proposed as universal helper T-lymphocyte and used as carrier for pneumococcal and *Shigella* antigens [43,44,45]. A synthetic peptide derived from polio virus was investigated as T cell epitope in a fully synthetic three-component anti-cancer vaccine containing a tumor associated glycopeptide [46]. Synthetic peptides derived from *Candida albicans* have been conjugated to a synthetic β-mannan trisaccharide and elicited in mice specific antibodies against the carbohydrate and peptide moieties. However, mice were immunized using the antigen-pulsed dendritic cell (DC)-based strategy which does not represent a suitable immunization protocol in standard clinical settings [47]. 

### 7.1. Proteins with Dual Role of Carrier and Antigen

As discussed above, the primary role of the protein carrier in glycoconjugate vaccines is to provide T cell epitopes that confer a T-dependent character to an otherwise T-independent antigen, such as capsular PS, and make the immune response to the saccharide component similar to the response to proteins. This task is clearly done by the protein carriers present in the currently licensed conjugate vaccines. Interestingly TT, DT and CRM_197_ based glycoconjugates elicit also antibodies against the respective related toxins (tetanus and diphtheria). PD is the carrier for eight out of 10 PS of the anti-pneumococcal conjugate vaccine PHiD-CV and was selected also with the aim to provide protection against NTHi acute otitis media [48]. The potential to benefit also from the protective antibodies elicited by the protein carrier of glycoconjugates has not been fully exploited until now. There is an increasing interest in investigating the dual role of the protein moiety of glycoconjugate vaccines as carrier a well as protective antigen, as this could simplify the formulation of multicomponent vaccines targeting both carbohydrate and protein virulence factors of a given pathogen. Accordingly, new carrier candidates from different pathogens have been investigated at preclinical level.

A genetically detoxified form of the pneumococcal toxin pneumolysin has been conjugated to pneumococcal PS, eliciting in mice specific antibodies against the related PS and also pneumolysin-specific IgG which neutralized pneumolysin-induced haemolytic activity in vitro [49]. 

*Staphylococcus aureus* derived recombinant proteins Hla, ClfB, and IsdB, conjugated to a synthetic non-acetylated oligosaccharide fragment of poly *N*-acetyl-d-glucosamine (PNAG), induced in mice specific IgG against the carrier protein with functional activity [50]. Wacker et al. showed that Hla bioconjugate with *S. aureus* type 5 capsular PS induced in rabbits and mice specific antibody titers against the glycan and the protein moiety both with protective activity [35]. When a fusion of two variants of meningococcal fHbp was conjugated to meningococcal ACWY PS, immunized mice developed bactericidal titers against the four meningococcus serogroups but also against serogroup X, indicating the potential role of the protein carrier as protective antigen too [51]. In a more recent work, Romano et al. conjugated recombinant *Clostridium difficile* toxin fragments TcdA_B2 and TcdB_GT to PSII surface PS from *C. difficile*. While only the TcdB_GT conjugate elicited in mice anti-PSII antibody titers comparable to those induced by a CRM_197_-PSII one, both conjugates induced anti-carrier antibodies with toxin neutralizing activity in vitro comparable to the non-conjugated proteins [52]. 

Group B Streptococcus pili proteins GBS80 and GBS67 have also been proposed as carrier for the capsular PS type II and V, respectively, from the same species [53,54]. Since there are three genetically distinct types of pili, namely PI-1, Pl-2a and Pl-2b, and more than five epidemiologically relevant serogroups bearing different capsular PS, conjugates combining protein and glycans can be seen as a means to reduce the vaccine complexity and broaden the coverage.

Simon et al. proposed flagellin as the carrier protein for *Salmonella Enteritidis* O-antigen, both being virulence factors and protective antigens, with the potential to achieve enhanced protection by the additive effect of anti-O-antigen and anti-flagellin immune responses [55].

### 7.2. Nanoparticle Carriers

Nanoparticle systems have been explored for the display of carbohydrate antigens, combining the multivalent presentation of carbohydrates with the special physico-chemical properties of nano-sized particles.

Keyhole limpet hemocyanin (KLH) (a large multisubunit metalloprotein found in the hemolymph of the giant keyhole limpet, *Megathura crenulata*), the Qβ virus-like particle and OMPC have been used as carrier for tumor antigens, the Alzheimer related peptide amyloid-β-peptide, and synthetic HIV associated glycans [7,56]. KLH has been also employed in the generation of a glycoconjugate targeting *B. anthracis* spores [57]. Short synthetic *S. pneumoniae* oligosaccharides have been coupled to Qβ virus-like particles, eliciting serotype specific, protective and long-lasting IgG antibodies of nanomolar affinity against the target glycans in mice [58]. Recently, Glycoengineered Outer Membrane Vesicles (geOMVs) have been designed for expression of pneumococcal PS, PNAG and *F. tularensis* O-PS [59,60,61,62]. Liposome presenting on the surface a pentadecasaccharide from the lipopolysaccharide of *Shigella flexneri*, a T cell peptide from influenza hemagglutinin (H307-325) and the immunopotentiator Pam3CAG, induced IgM and IgG titers against the native lipopolysaccharide [63]. In respect to liposome, geOMVs combine antigen presentation and the immunopotentiator effect of the TLR 2 and 4, naturally present on these systems. 

T cell peptides displayed on inorganic nanoparticles have been also tested as carrier for carbohydrate epitopes. Gold nanoparticles presenting a synthetic tetrasaccharide epitope related to pneumococcal type 14 PS, the T-helper ovalbumin 323–339 peptide (OVA323–339), and d-glucose elicited anti-PS IgG antibodies, albeit with lower bactericidal activities than the carbohydrate antigens conjugated to classic carrier proteins [64]. In a follow up work, this platform was used to expose onto the gold core the tetrasaccharide fragment from *S pneumoniae* serotype 14 and a trisaccharide fragment of serotype 19F (Tri-19F), combined with a T-helper peptide and d-glucose. Mice immunization showed that antibodies were exclusively elicited against the pneumococcal 14 oligosaccharide, probably due to interference with the other glycan [65].

## 8. The Chemistry of Protein Carriers

**Figure 1:**
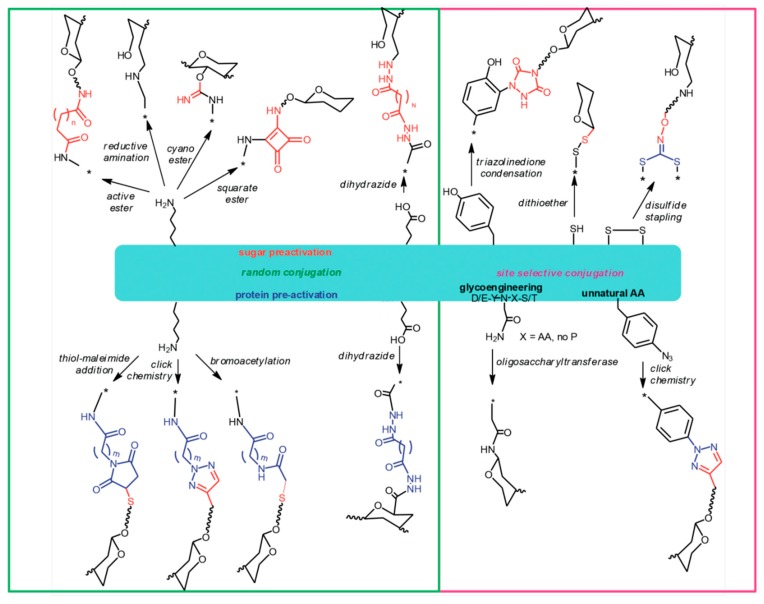
Different ways to attach glycans to protein carriers.

Carbohydrates can be linked to proteins applying a variety of approaches (Figure 1). Amino acid residues that are most suitable for chemical linkage to sugars are those who are exposed onto the protein surface and whose side chains have reactive functional groups, such as primary amino groups of lysines and carboxylic groups of glutamic or aspartic acid residues. Carboxyl groups of proteins can be conjugated to amino or hydrazido derivatives of sugars by a carbodiimide mediated condensation. Lysine amino groups can be coupled to sugars by reaction with their derivatives containing active groups such as succinimido esters or cyano-esters, squarate, or by direct reductive amination [7,66].

In some cases, the carrier protein is rendered more prone to conjugation, by incorporating spacers offering appropriate functional groups. The protein is typically modified introducing hydrazides, bromoacetyl groups or maleimido groups that react with carboxyl groups, cyano-esters or thiol groups introduced into the carbohydrate, or vice versa introducing sulfhydryl groups to react with bromacetyl or maleimido derivatized PS [67]. Azido groups can also be introduced into proteins for subsequent coupling to glycans derivatized with alkynes, via Copper (I) catalyzed or strain promoted cycloaddition [54,68,69]. Generally, linkage of a PS to a carrier protein results invariably in a random display of the carbohydrate onto the protein surface, although some selectivity can be obtained by modulating the carbohydrate to protein stoichiometry [70]. More selectivity has been achieved by targeting other amino acids such as cysteine [71], cysteine disulfide bridges or tyrosines [54,72], and using enzymatic chemistries [69]. 

A technology that allows selectively placing the glycosylation site on the carrier protein is currently emerging and is known as “bioconjugation”. In this approach, the carrier protein engineered to contain one or more *N*-glycosylation consensus sequences, the glyco-antigen, and an oligosaccharyl transferase are expressed in *E. coli* cells where the enzyme catalyzes the transfer of the saccharide chain to the asparagine residue of the *N*-glycosylation site [73,74,75]. Selective conjugation can be achieved also by introducing into the carrier protein sequence un-natural amino acids bearing functional groups for selective conjugation to glycans via click chemistry [76]. These approaches are of particular interest when the protein has dual role carrier-antigen, assuring the preservation of the key protein epitopes. In a new approach called Multi-Antigen Presenting System (MAPS), the carrier protein is fused to an avidin-like peptide which then couples with strong affinity to biotinylated PS [77]. Recently, PS entrapped in cross-linked protein [78], and liposomal encapsulation of PS and proteins [79] have been proposed, based on non-covalent association of PS with protein. 

## 9. Selection Criteria for Protein Carriers

As discussed above, the first proteins used as carrier for glycoconjugate vaccines were selected among those already licensed for human vaccines and with a strong track record of safety in conjunction with availability at industrial scale, such as TT and DT. Safety and large scale manufacturability are obviously key selection criteria also for new protein carriers as well as for all new vaccines intended for human use. Any toxic or enzymatic activity should be removed before testing a protein as new carrier. As learned from the history of tetanus and diphtheria toxoids, detoxification can be achieved by chemical treatment with formaldehyde, however this results in extensive modification and heterogeneity that renders more difficult product characterization and might limit the accessibility to some amino acid residues for conjugation.

Whenever possible, genetic detoxification, which selectively intervenes on specific amino acids, is preferred because of the well-defined structure and accessibility of sites for conjugation of the resulting toxoid. Typical examples are CRM_197_, recombinant Hla, *r*EPA and recombinant pneumolysine cited above.

The suitability of a new protein as carrier is subject to successful preclinical studies confirming the immunogenicity of their conjugates, preferably in comparison to a bench mark carrier.

An important requirement, shared with all antigens, is manufacturability at large scale and according to cGMP. Manufacturability means yields, production cost and quality of the product. A protein that is produced with low yields or whose manufacturing process is not viable at large scale in a cGMP environment, and does not meet the purity requirements (currently at least >90%) has limited probability of success. Quality implies also extensive knowledge of the protein physicochemical and immunological characteristics that are critical to ensure the reproducible production of the glycoconjugate vaccine itself. Protein carriers should contain a sufficient number of exposed amino acids targeted for the selected conjugation process, should be stable and have good solubility in the buffers and concentrations at which conjugation reactions take place. In the case of proteins engineered to insert one or more sites for saccharide attachment, these should be sufficiently surface exposed to allow efficient in vivo glycosylation or in vitro glyco-conjugation. 

In the case of the dual role carrier/protective antigen, the protein provides not only T cell helper epitopes, but also protective B cell epitopes; therefore, carrier selection and vaccine design become more complex and have to meet additional criteria as compared to the classical carrier role. Epitope mapping of the carrier protein to inform the design of the conjugation is critical to avoid loss of protective epitopes on the protein following conjugation.

## 10. Characterization of Protein Carriers

Proteins such as TT and DT are produced and licensed as standalone vaccines and are required to meet the corresponding World Health Organization and pharmacopeia requirements.

Depending on the manufacturing process, DT and TT purified bulk preparations can show different degrees of purity. Typically, the antigenic purity, as determined by the flocculation test (in vitro assay in quality control), should be at least 1500 Lf (limit of flocculation) units/mg protein for DT and 1000 Lf for TT. 

Additional characterization may be considered to monitor multimerization or aggregation and purity using size-exclusion chromatography-high performance liquid chromatography (SEC-HPLC) coupled with light scattering detector [80] and sodium dodecyl sulfate-polyacrylamide gel electrophoresis (SDS-PAGE). 

For the other protein carriers used in licensed conjugate vaccines (OMPC, CRM_197_ and PD), analytical characterization and release tests panels have been defined and agreed with Regulatory Agencies during the development of the respective conjugate vaccines. The purity of CRM_197_ batches is expected to be >90%, and often >95%, as determined by HPLC, SDS-PAGE or capillary electrophoresis (CE). CRM_197_ contains an exposed loop of 3 arginine residues that is clipped by proteases present in the culture medium, resulting in a “nicked form” where subunits A and B are still linked through a disulfide bond. The intact polypeptide and the two fragments A (~21 kDa) and B (~38 kDa), derived by proteolytic clipping followed by dithiothreitol reduction, can be easily detected by SDS-PAGE in reducing conditions [26]. The manufacturing process is expected to produce CRM_197_ with a consistently low degree of nicking, generally <5% of total CRM_197_. As mentioned above, CRM_197_ is now available through recombinant DNA techniques which provide a product highly comparable to that obtained from *Corynebacterium diphtheriae* platform [25].

The analytical characterization of new protein carriers for glycoconjugate is similar to that of other protein antigens included in vaccines for human use. Most of the potential new carriers are recombinant proteins produced in *E. coli*. As discussed above, an extensive knowledge of the protein physicochemical and immunological characteristics is important to ensure the reproducible production of the glycoconjugate vaccine itself. A typical initial characterization panel for these antigens is reported in Table 1. A restricted list from this panel might be included into the routine quality control tests for release, once sufficient knowledge of the protein biophysical and immunological characteristics has been acquired and process consistency has been demonstrated. In the case of process changes or transfer technology to a new manufacturing site, such knowledge is used to build a panel of characterization tests and related specifications to ensure comparability with historical batches.

If the carrier is meant to also play the role of antigen, eliciting functional antibodies, it is important to know where the key B epitopes are located in the protein topography to preserve them during the conjugation. Key reagents to map the protein epitopes are monoclonal antibodies, in particular functional ones. Techniques such as X-ray crystallography, Hydrogen Deuterium Exchange Mass Spectrometry (HDX-MS), peptide scanning and phage display can be used to characterize, with different accuracies, the epitopes recognized by a particular monoclonal antibody [81,82,83]. Surface Plasmon Resonance (SPR) should be used for providing affinity values of the antibody-antigen interaction [83]. 

### 10.1. Characterization of Derivatized Protein Carriers

As mentioned above, depending on the chemistry chosen, protein carriers might be derivatized before conjugation to PS. The derivatized protein carrier should be characterized with methods suitable to detect and quantify the derivatization degree. The extent of derivatization should be controlled to avoid over-derivatization that might mask potential T cell epitopes of the carrier protein.

For example, the extent of hydrazide derivatization of proteins can be determined using colorimetric assays [84], but also by MS Q-TOF or MALDI-TOF analysis [85]. Thiols groups introduced into protein carrier can be measured by the Ellman’s reagent for free thiols [86]. 

For protein carriers genetically derivatized to insert glycosylation consensus sequences or un-natural amino acids, the incorporations can be confirmed by sequence analyses, amino acid analysis, and mass spectrometry or by in vitro fluorescence labeling techniques [76,87].

Table 2 reports a list of analytical methods for the control of derivatized protein carrier.

### 10.2. Characterization of the Conjugated Protein

Following conjugation, the protein carrier is modified at one or more sites. Depending on the coupling strategy, different glycoconjugates configurations are possible: (a) monomeric or limited cross-linked glycoconjugates, where the protein carrier is modified with multiple saccharide chains that have one or more activation sites for conjugation; and (b) cross-linked conjugates where multiple activated saccharide chains and protein molecules couple together creating a cross-linked network.

In the case of monomeric glycoconjugates, from the protein to PS ratio (*w*/*w*) and the molecular weights of both protein carrier and glycan, it is possible to calculate the average number of saccharide molecules attached to the protein. When reductive amination was used as chemistry for the Hib conjugate vaccine, a method based on complete removal of the carbohydrate moiety resulted in a novel stable derivative of the lysines previously conjugated, and its quantitative determination by amino acid analysis provided the degree of conjugation independently from the glycan analysis [88]. 

However, this information does not tell us which amino acids of the protein have been involved in the conjugation reaction.

For a comprehensive characterization of the glycoconjugates and for comparability assessing following process changes or technological transfer to other manufacturing sites, the availability of methods for determining the conjugation profile of the protein carrier is very important. Mass spectrometry is a potent tool for determining the location of the saccharide chains on protein carriers. For long and heterogeneous PS, the peptide mapping procedure can be modified to include a step for carbohydrate moiety removal prior mass spectrometry analysis [89,90,91]. 

Table 3 summarizes main assays for protein moiety characterization of glycoconjugate vaccines.

## 11. Conclusions

The protein component of glycoconjugate vaccines actively participates in their immunological processing providing T cell epitopes that are critical for the induction of high avidity antibodies in all classes of age, including infants, and for eliciting a memory response. 

Licensed glycoconjugate vaccines mostly use traditional carriers. DT, TT and CRM_197_ have been used for Hib, pneumococcal and meningococcal conjugate vaccines. Exceptions are constituted by OMPC and PD, used, respectively, by Merck and GSK for their Hib and pneumococcal conjugate vaccines.

From the clinical studies on glycoconjugate vaccines, it is in general difficult to draw conclusions about which protein carrier has the better impact on conjugate vaccine immunogenicity. This is because, besides the carrier, and for a given carbohydrate, there are other parameters that can influence the immunogenicity of glycoconjugate vaccines such as conjugation chemistry, the presence or not of a spacer, saccharide size and degree of conjugation [7].

There is a flowering of new protein carriers under evaluation which is driven by several considerations.

For example, pre-exposure or co-exposure to a certain carrier can lead to reduction of the anti-carbohydrate immune response against glycoconjugate vaccines, via immune interference phenomena called carrier epitope suppression and bystander effect [92]. The use of more and more composite immunization schedules increases the likelihood of immune interference. The investigation on new carriers is driven also by the interest to explore the dual role as carrier and protective antigen that a pathogen related protein can play, thus resulting in a vaccine that, by simultaneous administration of carbohydrate and protein antigens, tackles two different virulence factors of the pathogen. In this case, the extent and location of the saccharide chains on the protein carrier might be relevant in terms of preservation of its key B-cell epitopes. The availability of analytical techniques to map both protein and carbohydrate epitopes become important as well as methods to determine the protein sites that have been conjugated and to assess the impact on protein folding. Site specific conjugation chemistries or glycoengineering, which deliver products with predefined connectivity of the carbohydrate to the protein, are powerful tools to prevent any possible detrimental effect of glycoconjugation on the protein immunogenicity and to simplify the quality controls needed for vaccine release. 

Synthetic peptides, either directly conjugated to the glycan or linked to a liposome or a gold nanoparticle core, have been proposed as alternative carriers [42,43,44,45,46]; the biggest challenge for human application is the MHC class II genetic restriction which requires a diversity of T cell epitopes presented by proteins to better address its genetic polymorphism. 

Novel carrier systems, in particular nanoparticles, have been recently proposed as alternative to traditional proteins. Presentation of multiple copies of carbohydrate antigen (*multivalence*), favoring B-cell activation, and in a conformation that resembles that on native bacteria, joint to optimal nanoparticle size for immune stimulation, seem very promising to drive an effective immune response. Among nanoparticles, OMVs combine antigen presentation with intrinsic adjuvant properties [93] and are receiving great attention.

The selection of new protein carrier is guided by a matrix of factors which include manufacturability at industrial scale joint to the appropriate grade of purity to meet the quality requirements needed for clinical testing and for future commercial supply. Ability of the protein to provide T cell help and increase the effectiveness of the conjugated PS is of course fundamental. 

Glycoconjugate vaccines can be produced with a variety of methods. The question whether the connection site of the saccharide chain to the protein might impact on the conjugate immunogenicity has no clear answer at the moment. Stefanetti et al. prepared *Salmonella Typhimurium* O-antigen conjugates, characterized for having one only sugar chain linked to different amino acids on CRM_197_ and noticed that the induced immune response was different depending on where the saccharide chain was located [69]. In the context of mouse immunization studies with synthetic β-glucan oligosaccharide–CRM_197_ conjugates, it has been found that four modified tyrosines can elicit anti-glucan IgG titers not significantly different from those induced by higher levels of glycation at lysine of CRM_197_ [94]. Similar studies should be extended to different PS and carrier proteins, to better understand the impact of conjugation site on immunogenicity.

In terms of number of linkage sites per protein molecule, Pozsgay et al. observed that a high level of protein modification could be needed for optimal immune response of short synthetic *Shigella dysenteriae* type 1 oligomers, while a lower modification level could be sufficient for longer oligosaccharides, and extensive modification could result in lower response due to masking of T cell epitopes of the protein [95]. This might suggest the need of a right balance between number and length of saccharide chains attached to the protein carrier and the preservation of the protein T cell epitopes. 

Traditionally, the covalent linkage of PS antigens to the carrier protein is thought to be fundamental to the immunological properties of conjugate vaccines. Recently, there is evidence, in animal models, that covalent attachment to carrier proteins might not be required for conversion of T-independent antigens into T-dependent ones. High affinity non-covalent interaction between carrier and PS such as biotin–avidine based conjugates [77] and different co-presentation systems such as PS entrapped in cross-linked protein matrix [78] and liposomal formulations [79] are under investigation. 

These new approaches, together with the use of novel carrier systems [64,93], also appear interesting to further investigate and better understand the mechanism of action of glycoconjugates, supporting the design of improved vaccines. Promising results obtained in animal model need to be confirmed in humans, especially in infants, and the ability of these systems to convert T-independent carbohydrate antigens into T-dependent ones, in comparison to traditional glycoconjugates, needs to be assessed.

## Figures and Tables

**Table 1:** Typical characterization panel for protein carriers before conjugation.

Category	Attribute Measured	Key Techniques	Comment	Impact on Glycoconjugate
Purity	% of target protein	SDS-PAGE, SEC-HPLC, RP-HPLC	Impact on consistency, potency and safety	Low purity might reflect in poor glycoconjugate purity
	Truncated or degradation forms	SEC/RP-HPLC, MS, ELISA	Product-related substances or impurities (depending on retention of biological activity)	Heterogeneity of glycoconjugates which might impact on consistency and immunogenicity
	Aggregation	SEC-MALS, DLS, MS	Aggregation is source of heterogeneity: impact on manufacturing consistency, immunogenicity, and safety	Heterogeneity of glycoconjugates which might impact on consistency and immunogenicity
Impurities	Host cell residual proteins	WB, ELISA, MS	Poor control of process related impurities might impact on consistency, potency and safety	Poor control of process related impurities might impact on consistency, potency and safety
	DNA	Picogreen, Threshold		
	Chemical residuals	HPLC, Colorimetric assays, NMR		
Contaminants	Endotoxins, Sterility or Bioburden	LAL, Microbial count, Compendial sterility test	Impact on safety	Impact on safety
Identity and Structure	Antibody binding	WB, ELISA	Immunochemical identity	Important if dual role of carrier and antigen is considered
	Key Protein B cell Epitopes	Epitope mapping tools: X-ray, HDX-MS, SPR	Mapping of key functional protein epitopes	Important to precisely map key protein B cell epitopes if dual role of carrier and antigen is considered
	Primary structure, Intact Mass, Amino acidic composition	MS, Amino acids analysis	Classical physicochemical protein identification tests	Well defined protein moiety of glycoconjugate
Structure	Secondary structure	CD	Structure knowledge important if dual role of carrier and antigen is considered	Important to compare structure before and after conjugation if dual role of carrier and antigen is considered
	Tertiary structure	FLR, DSC, X-ray, EM		
	Lipidation, Deamidation, *N*-terminal methionine, Glycation	MS, SDS- PAGE, WB, ELISA, CE	Post-translational modifications	Lipidation might provide adjuvant effect; deamidation might results in additional conjugation sites (depending on the chemistry); glycation might interfere with carbohydrate analyses on conjugates
Stability	Purity, Integrity, Identity, Sterility/Bioburden	SDS PAGE, SEC-HPLC, RP-HPLC, SEC-MALS, WB, DLS	Stability protocols at different temperatures are practices common to all biological products	Impact on glycoconjugate stability
Toxicity	Safety	In vitro tests, animal studies	Standard for vaccines	Standard for vaccines

Legend: SEC-HPLC: Size exclusion chromatography-HPLC; RP-HPLC: Reverse phase-HPLC; MS: Mass Spectrometry; ELISA: Enzyme Linked Immunosorbent Assay; SEC-MALS: Size exclusion chromatography coupled with Multi Angle Light Scattering; DLS: Dynamic Light Scattering; NMR: Nuclear Magnetic Resonance; LAL: Limulus Amebocyte Lysate; HDX-MS: Hydrogen Deuterium Exchange Mass Spectrometry; SPR: Surface Plasmon Resonance; CD: Circular Dichroism; FLR: Fluorescence Spectroscopy; DSC: Differential Scanning Calorimetry; CE: Capillary Electrophoresis.

**Table 2:** Example of characterization approach for derivatized protein carriers.

Category	Attribute Measured	Key Techniques	Comment	Impact on Glycoconjugate
Structure	Extent of chemical derivatization	MS and/or colorimetric assays suitable for the kind of derivatization (e.g., hydrazide, thiol)	Level of chemical derivatization can inform conjugation stoichiometry. Can impact on protein folding, protein T and B cell epitope preservation and aggregate formation	Level of chemical derivatization dictates the maximum protein to carbohydrate ratio, and immunogenicity
	Site of chemical derivatization	MS	Can impact on protein epitopes	Informs the conjugate structure and impact on immunogenicity
	Genetic derivatization	DNA Sequence, MS, in vitro labeling techniques	Glycosylation sequences or un-natural amino acids amenable for conjugation can be inserted into the carrier protein	Precisely inform the glycoconjugate structure
Purity	Aggregation	SEC-MALS, DLS, MS, SDS-PAGE (covalent aggregates)	Chemical derivatization can cause covalent or non-covalent aggregation with consequent glycoconjugate heterogeneity	Heterogeneity of glycoconjugates might impact on consistency and immunogenicity
Identity	Antibody binding	WB, ELISA, SPR	Mapping of key functional protein epitopes	Important to preserve key protein B cell epitopes during conjugation if dual role of carrier and antigen is considered

**Table 3:** Example of characterization panel for the protein moiety of glycoconjugate vaccines (drug substance).

Category	Attribute Measured	Technique	Comment
Proof of conjugation (potency)	Covalent linkage between protein and glycans	SDS PAGE, WB, MS, SEC-HPLC	Covalent linkage is the gold standard for glycoconjugate vaccines
Extent of conjugation	Protein to Carbohydrate ratio	Protein content in conjunction with sugar quantification: HPAEC-PAD, colorimetric assays, Mass Spec	Mass Spec is applicable only to conjugates with well-defined synthetic carbohydrates
Identity	Antibody binding	WB, ELISA, SPR	Mapping of key functional protein epitopes
Identity and Structure	Key protein B cell epitope	Epitope mapping tools, X-Ray, HDX-MS, SPR, in vitro potency assays	Key protein B cell epitopes should be preserved if dual role of carrier and antigen is considered
Structure	Location of saccharide chains	MS	Confirmatory for site selective conjugation methods and glycoengineering methods where attachment sites are predetermined
Structure	Secondary and tertiary structure	CD, FLR, DSC	Secondary structure can be modified as result of glycoconjugation
Potency	Free protein	SDS PAGE, SEC-HPLC, CE	Levels of un-conjugated protein might interfere with glycoconjugate immunogenicity
Purity	Aggregation and multimerization	SDS PAGE, HPLC, SEC-MALS, DLS	Heterogeneity of glycoconjugates might impact on consistency and immunogenicity
Stability	Purity, Integrity, Identity, Sterility/Bioburden, Free protein	SEC-HPLC, SEC-MALS, SDS PAGE, WB	Generally part of the stability program for the glycoconjugate antigen

Does not include tests for the carbohydrate moiety of the glycoconjugates and other standard tests routinely done for vaccines such as process impurities, sterility/bioburden, and LAL.
